# Why do women not deliver in health facilities: a qualitative study of the community perspectives in south central Ethiopia?

**DOI:** 10.1186/1756-0500-7-556

**Published:** 2014-08-21

**Authors:** Meselech Assegid Roro, Emebet Mahmoud Hassen, Alemayehu Mekonen Lemma, Seifu Hagos Gebreyesus, Mesganaw Fantahun Afework

**Affiliations:** Department of Reproductive health and health service management, School of Public Health, College of Health Sciences, Addis Ababa University, P.O.Box 28287/1000, Addis Ababa, Ethiopia; Center for Population Studies, Addis Ababa University, Addis Ababa, Ethiopia

**Keywords:** Qualitative, Delivery, Skilled attendant at birth, Community perspective, Ethiopia

## Abstract

**Background:**

In Ethiopia most childbirth occurs at home and is not assisted by skilled birth attendants. On the other hand having a birth attendant with midwifery skills during child birth is one of the most important interventions in reducing maternal morbidity and mortality. The objective of this study was to make an in-depth assessment of reasons why mothers do not use health facilities for child delivery.

**Methods:**

Focus Group Discussions were used to gather information on use of health facilities for delivery in Butajira districts of South Central Ethiopia. The study was conducted from January to February 2012. Information was collected from four groups of women who had delivered in the past two years and four groups of men whose wives/partners have delivered in the same period. Data was coded and categorized using open code, qualitative data management software and analyzed based on thematic analysis.

**Results:**

A total of eight FGD sessions, four with women and four with men groups were conducted involving 81 residents of the Butajira district. FGD participants answered that a large majority of women in the district gave birth at home. Two major themes, client related factors and facility/staff factors, emerged. Factors that emerged within major themes of client factors were decision making on place of delivery, reliance on Traditional Birth Attendants (TBAs), misconception about services provided at health facility, inability of family members to be present at time of labor and delivery, lack of privacy, traditional and/or spiritual factors, economic factors and accessibility to health care facilities. Within major themes of facility/staff factors subthemes that emerged were poor reception, refusal of admission, lack of privacy, information gap, poor competence and shortage of staff and materials at health facilities.

**Conclusion:**

Women in the study areas do not deliver in health facilities because of reasons that can be attributed to health care system and client related factors. These need to be addressed by considering the specific factors related to the health system and community perspectives.

## Background

Having a health worker with midwifery skills present at child birth is one of the most important interventions to reduce maternal morbidity and mortality [1]. Considering this fact, the proportion of births attended by skilled health personnel is used as one of the major indicators to monitor progress towards the achievement of the Millennium Development Goal of reducing maternal mortality ratio [2–4].

According to the definition of World Health Organization (WHO) a skilled birth attendant (SBA) is defined as "an accredited health professional such as a midwife, doctor or nurse- who has been educated and trained to proficiency in the skills needed to manage normal (uncomplicated) pregnancies, childbirth and the immediate postnatal period, and in the identification, management and referral of complications in women and newborns" [5, 6].

As barriers to accessing skilled attendance during delivery, women in sub-Saharan Africa encounter sociocultural factors, perceived benefits, economic accessibility and physical accessibility [7]. Women in sub-Saharan Africa also face limited access to skilled delivery, especially in the rural areas [8, 9].

In a study done in Malawi, Kumbani et al. reported that onset of labor at night, short labor, rainy season, and health workers attitudes were significant barriers for the women to deliver in a health facility when labor started. Socio-cultural factors were also related to women delivering at home. They recommended further exploration of barriers that prevent women from accessing health care for better understanding and subsequently identification of optimal solutions with involvement of the communities themselves [10].

Ethiopia has one of the highest maternal mortality ratios in the world with 676/100000 live births and about 90% of child births occur at home and are not assisted by skilled birth attendants [11]. To improve the current situation of utilization of skilled attendant at birth, the government of Ethiopia has designed a number of policies and strategies of which increasing accessibility and availability of services at the community level is one [12]. Moreover, the Health Sector Development Plan (HSDP) IV has set targets that include increasing deliveries attended by skilled attendants from 18% to 60% [13, 14]. According to EDHS 2011 the reasons reported for not using health facility for delivery by respondents were health facility delivery was not necessary (61%), it was not customary (35%) and health facility was either too far or that they did not have transportation (14%) [11]. As husbands are the heads of households, most of the decisions are mainly made by men and they control household expenditure in Ethiopian community. Thus men may have crucial role in the access of mothers’ to timely and skilled care. However, EDHS did not involve male partners and didn’t also address health professional related factors. Moreover, it also did not explore the details of the client related factors. Thus the objective of this study is to make an in -depth assessment of the reasons why mothers do not use health facilities for child delivery in the district of south central Ethiopia.

## Methods

### Study setting

The study was conducted in Butajira, Southern Nations, Nationalities and People’s Region (SNNPR) South Ethiopia. These study area was purposely selected because the region is home to a University that is currently being mentored by Addis Ababa University (AAU) under the framework of the Gates Institute, Johns Hopkins University, Bloomberg School of Public Health – Addis Ababa University collaboration. Butajira district also includes a world renowned health and demographic surveillance site (Butjira Rural Health Program) [15] which provides a fertile ground for community based research

### Study design

Focus Group Discussions (FGDs) were conducted among women and men from urban and rural areas of the study district. The number of people involved in the discussion was decided to be 8–12 following the agreed standard number of FGD participants. Selection of participants was made in consultation with local village leaders based on the following criteria: women who delivered in the past 2 year and men whose wives/partners delivered in the same period, residents of the local village, knowledgeable of the maternal health situation in the area and who could express their opinion and discuss freely.

### Data collection

First an interviewer guide was developed in English. It was then translated to the Amharic language and translated back to English. Data collection was done during the month of January-February, 2012. Interviews were conducted in a quiet office and privacy was ensured to enable discussants to feel free and express their opinions. These interviews were conducted for female and male separately in both urban and rural settings. Besides taking field notes on a face to face discussion, all FGD sessions were audio taped. The sessions took about one and half hours on the average.

Focus Groups Discussion was chosen rather than some other qualitative methods as group dynamics and interaction was thought to create better anonymity and spontaneity. The combined effect of the group produced a wider range of information, insight and idea than number of individuals’ responses [16].

The FGDs were conducted by skilled moderators who had second degree in public health and social sciences selected based on previous experience of conducting qualitative studies. The female team member of investigators and a female research assistant conducted the FGDs for women and a male team member investigators and a male research assistant conducted the FGDs for men. A comprehensive training and updating on the overall objective and procedures of the study and moderating skills was given for two days for the study team by the principal investigator.

### Data analysis

Tape-recorded data were transcribed and extensive notes of interviewers were reviewed. All Focus Group discussions were conducted in Amahric language then translated in to English and back translated to Amahric. Data was entered and analyzed using Open Code software for qualitative data analysis [17]. Thematic analysis was used. First open (crude) codes were created by reading line by line of the verbatim transcript. In the next step selective coding was performed and relevant codes were further categorized to form thems. Prominent themes were then further categorized to subthemes. Verbatim quotations were frequently used to illustrate the responses of the respondents on important issues and sub themes.

### Ethical considerations

Ethical clearance was obtained from Institutional Review Board Addis Ababa University College of Health Sciences. Additionally, permissions were obtained from relevant Federal and local government offices and informed verbal consent was obtained from all focus group discussants. Verbal consent was preferred to written consent as many of the study participants were illiterate.

Risk from the study procedure to the study participants was minimal. The consent form was read by the moderator before each focus group discussion and with the consensus of the study participants, verbal consent was voice recorded.

## Results

A total of eight FGD sessions, four with women and four with men groups were conducted. A total of 81 (36 men and 45 women from Butajira) residents of the district participated in the study. All participants agreed that a large majority of women in the district give birth at home. The participants identified multiple factors that hindered the utilization of health facility for delivery which can be classified in to two major themes. The themes were client related factors and facility/staff related factors.

The major theme of client factors included the following subthemes: decision making on place of delivery, reliance on Traditional Birth Attendances (TBAs), misconception about the services provided at health facility, inability of family members to be present at time of labour and delivery, lack of privacy, traditional and/or spiritual factors, economic factors and accessibility to health care facilities.

Within the major theme of facility/staff related factors the subthemes that emerged were poor reception, refusal of admission of laboring mother at health facility, lack of privacy, information gap, and poor competence and shortage of staff and materials at health facilities. The subthemes that relate to the two main themes are described below.

### Client related factors

#### Decision making on place of delivery

It was mentioned that decision making about place of delivery might be initiated by the woman or the husband. The primary decision maker was said to be the husband by most participants in Butajira community. This was particularly true for a woman from rural community.

*"No woman decides by herself on place of delivery except the husband "…. Male participant from rural Butajira*

### Reliance on TBA care

One of the client related factors identified as a reason for not seeking health facility delivery was related to reliance on TBA care in the Butajira community.

*"Pregnant women hope that the traditional birth attendants are with them to assist during labor and delivery. Women also depend on them." ………Male participant from rural Butajira*

Laboring mothers were frequently noted to have been cared for by TBAs for days before transfer to health care facility. The participants also reported incidents at which laboring mothers were retained by the TBAs even after complications developed.

One participant from Butajira expressed the following:

*"I know one mother who died 4 months back. She was in labor for two days being attended by traditional birth attendant. While in labor she started to bleed which the TBA tried to stop but couldn’t. The woman went to hospital too late with massive bleeding. She died in the hospital two hours after delivery"……..Female participant, from urban Butajira*

Women trust TBAs, the TBAs also advice mothers to deliver at home and be attended by them. This continues to be the norm even nowadays.

### Misconception of services provided at health facility

Focus Group Discussion (FGD) participants believe that every woman who delivers at a health facility undergoes either minor or major operations such as genital cutting or stitching (episiotomy) or cesarean section. And thus, women avoid giving birth at health facilities.

*"A mother who gave birth at home eight times without health problems went to hospital to deliver on her ninth pregnancy. She was operated at hospital, which showed that health professionals hurry to undertake operation and stitching"……*

*Male participant, from urban Butajira*

Moreover, women frequently mentioned that once the genitalia is cut it remains open and the problem will be there forever. Most women participants did not consider service utilization at health facility as having any advantage. They prefer to pray to Allah/God rather than go to health facility.

When cases which needed further management and care were identified at the health center and they were advised or referred to hospital, they went back home and called TBAs for delivery.

*"I went to health center and they referred me to hospital by diagnosing narrow pelvis. I then went back home and called TBA and then I delivered after 2 days of labor"*……*Female participant from rural Butajira.*

### Inability of family members to be present during labor and delivery in health facilities

Most FGD discussants agreed that one of the reasons why women prefer to give birth at home is that it is not allowed to have somebody with laboring mother at hospital or health center. Normally, a woman in labor is supported physically and psychologically when she is at home. Relatives would be with her to support, encourage her and pray for safe delivery. They also feel that after delivery the new born is protected better from cold at home since it is well handled by the family.

### Traditional and/or spiritual factors

The participants said that home delivery is almost a traditional practice and custom and women will not go to a health facility for delivery except at times of problems. Even those who attend ANC at health facility stay at home at the time of labor and delivery praying so that God/Allah helps them since He is the one who knows and decides on the outcome. There is also a widespread belief that it is St Mary that helps in smooth child bearing and protection even if women deliver at home.

Generally the community does not see any problem related to home delivery. A reason why women sometimes went to health facility was prolonged labor: when labor takes two to three days. Some of the participants said there is lack of understanding about the importance of delivering in health institutions in the community.

Regarding relationship of place of delivery and previous pregnancy the participants in both urban and rural Butajira mentioned that women who had already given birth commonly give birth at home than women who are pregnant for the first time.

*"My aunt who lives in town has four children and she attended ANC during all of her pregnancy at hospital and she delivered all of them at home"….Female participant from urban Butajira.*

*"I delivered seven children, all of them at home". … Female participant, from urban*

### Economic factors

Most of the participants mentioned that women whose families do not have money for transportation and service cost including cost of various treatments such as surgical operations prefer to stay at home during labour and delivery and be attended by Traditional Birth Attendant. Even those who know the importance of institutional delivery do not seek skilled attendants due to financial problem. Women also prefered to stay at home to save the money for their family rather than using it for delivery at health institution. This also lead to delay in seeking a skilled attendant as early as possible.

*"When pregnant mother wants to deliver at health institution it costs her $17 to $22. However, if the woman delivers at home it costs her only $0.8 for TBA"……Male participant from rural Butajira.*

A husband who encouraged and supported his wife for ANC follow up may hide himself or disappear during labour and delivery due to financial problem; money required for institutional delivery.

*"The payment is more than what we can afford. To a minimum one is required to pay $56. To do this it is expected from us to sell our land or borrow money from others. Due to fear of that the men disappear when their wife goes into labour "……Male participant from rural Butajira.*

FGD participants also agreed that laboring mothers prefer to give birth at health center than hospital because the service cost in health center is free or less when compared to that of hospital.

### Accessibility to health care facilities

Inadequate transport facility was also identified as a reason that contributed to home delivery. This was mainly related to poor road and lack of ambulance for transportation. In some cases health facility is far from the community. The common transportation mechanism in Butajira is horse cart. Additionally there are three wheel drive automobiles locally called ‘Bajaj’ in town. Both are not comfortable for laboring mothers.

In many rural areas, laboring mothers are transported to health facilities by locally made stretchers and most women choose to give birth at home than being carried on such a stretcher.

### Facility/staff related factors

#### Poor reception and inappropriate handling of laboring mothers who visit health facilities

According to the participants the reception of labouring mothers by health service providers is not welcoming and attractive. She does not get the proper care. Those women who previously gave birth at health facility do not advice others to go.

A man remarked "A woman in labour once came to a hospital while I was there with my sister. Soon after arrival, she started to deliver a baby while on a hoarse cart. Someone screamed ‘ …*I see a baby…she is giving birth!’ But none of the nurses showed up for help. The woman gave birth with the help of the mothers around her"*

*"They do not properly manage or handle a woman suffering in labour. So we become angry and think it is better to get the service of traditional Birth Attendants"…..Male participant from rural Butajira*

FGD discussants repeatedly mentioned that health service providers refuse to admit mothers in labour when they go to health facility. The reason given is "it is too early for admission". When mothers are sent back home and advised to come when labour progresses, theydo not go again and thus give birth at home.

*"Nine months ago when my wife was in labour I brought her to hospital for delivery. Health professionals assessed her and told me that she has to go back home and come back when her labour progresses. Then we went back home and she gave birth at home one hour after we left the health facility"…..Male participant from urban Butajira.*

### Lack of privacy

Lack of privacy is another concern raised by the participants. Participants reported that women are not comfortable when they expose their genitals to be examined by health professionals. They are particularly uncomfortable and feel embarrassed with vaginal examination.

*"I do not want to go to health center unless I have problem. They insert their fingers in to my genitalia. I hate that"…. .Female participant from urban Butajira*

The number of health care providers attending the labour also makes the laboring mother embarrassed. When eight or ten professionals come around, it is difficult for laboring mother. They are not comfortable to expose their genitalia when such number of professionals are around and particularly when exposed for repeated vaginal examination.

*" For a woman there will be eight to ten professionals to come around and attend her which is troubling for laboring woman to expose her genitalia"…….Female participant from rural*

### Information gap

The participants repeatedly mentioned that the only reason a laboring woman went to health facility for delivery was for complicated labour. This indicates that information given during pregnancy when women went for ANC was limited. They did not have any information regarding advantage of health facility delivery and birth preparedness plan.

*"I have three children and delivered all of them at home. No one asked/told me where I want/have to deliver. Health providers told me as I finished my ANC follow up that I don’t need to go again because I have finished my follow up. Then I stayed at home and when labour started I called TBA and delivered at home." ……Female participant from rural Butajira*

### Poor competence of health care providers

Participants gave examples why they believe health care providers are not competent. One example is delivery of placenta which leads to severe bleeding as a consequence of failure to do so. They also mentioned that service providers, either trainees or staff, do not have supervisors who regularly inspect their activities and gives feedback.

Women discussants in one of the groups commented on the role and responsibility of HEWs that they were not capable of assessing, identifying and referring women with problems to a health facility while these are their expected roles and responsibility. Some participants pointed out those health service providers, particularly nurses, who provide inappropriate care and harass laboring mothers verbally.

### Inadequate resources

Men participants said that lack of medical equipment in health posts and other facilities forces mothers to be attended by TBAs. On top of this, mothers prefer to be delivered by TBAs because health posts do not have enough drugs and equipment to provide the services and do not have a place where laboring woman can be admitted.

## Discussion

The study gives in-depth insight into a range of client and health facility/staff related factors for low utilization of health facility for delivery.

According to the Focus Group participants’, majority of women gave birth at home and home delivery is taken as a common practice in Butajira. . This finding is similar to the EDHS which reported that 6.1% of deliveries are conducted at health institution in SNNP region [11].

This study indicated that women relied on Traditional Birth Attendants for delivery which is related to various reasons including trust of TBAs and dissatisfactions with the health system. Because of this women arrive at health facilities after being in labour for very long time and ending up in severe complications. After they make the decision to go to health facility, more time of delay occurs in reaching to the health facility due to lack of transportation or lack of infrastructure. This finding is in agreement with other studies which reported that communities in rural areas prefer traditional birth attendants during delivery because of cheaper services, easy availability and cultural sensitivity [18]. Moreover lack of information about danger signs, and inaccessibility to health facility are the other reasons for delay of mothers’ arrival at the health facility [19–24].

That a decision to seek delivery care is mainly made by men in Butajira community may have consequences in getting timely and skilled care. Moreover, men control large household expenditure. They don’t have the knowledge of complications during pregnancy and childbirth and the need for early referral. These factors add to the delays in getting proper care. This is similar to the findings of study done in Nepal where men are the primary decision makers and a study in Ethiopia where place to give birth is decided by a man or husband [19–22].

Economic factors such as money for transportation cost and service cost is the other client reason for home delivery. This is similar to a study done in Ouargaye and Diapaga district , Burkina Faso and Southern Ethiopia were economic issue was one determining factor for home delivery [20, 25–27]. This is also similar to findings from EDHS which reported that one of the reasons for home delivery was lack of transportation. [11].

Our study findings also indicate that inability of family members to be present during labour and delivery in health facilities at health institutions is the other important reason for preference of home delivery. Traditionally, relatives are expected to be with the laboring mother to support, encourage her and pray for safe delivery. This finding is similar to the results of other studies [18, 20–22, 28, 29].

Other factors which we found influential for low use of health facility for delivery are related to the health facility and staff. It is generally related to their experiences of poor reception; refusal of admission of laboring mothers by the health workers which other studies have also witnessed [19, 20, 22, 25, 26].

Lack of privacy at health institutions was also mentioned by the community as one reason for preference of home delivery. The community lacks confidence in the capability of health facility staff to diagnose and provide correct management. The community’s perception of health facility staff poor competence such as misdiagnosis, mismanagement, misconduct, poor commitment, and poor care was equally important. Inadequate resources such as shortage of staff and materials at health facilities were additional factors mentioned by the participants of this study. Studies in Nepal, Burkina Faso and Ethiopia also showed similar findings [19, 26–28].

The strength of this study is that it assessed reasons why women do not use health facility for delivery in depth compared to other studies done in the study area. However, the results of the study should be interpreted cautiously. The site was selected purposively. And the country has diverse culture and different rates of skilled birth attendants. Even though the findings are similar with other studies, we cannot generalize the findings of this study for the whole nation.

## Conclusion

Client and facility/provider related themes emerged as contributors of low use of health facility for delivery.

Strengthening mobilization and awareness creation of the community about advantage of health facility for delivery is very important. At the same time improvement of family’s awareness and promotion of women decision making in maternal health service utilization has a great contribution. Accesses to health facility and availing ambulance for delivery in emergency situations are also important. A strategy has to be designed for better use of HEWs in referral of laboring mothers and facilitating their reaching to the health facilities. Moreover, the strategy should motivate and encourage TBAs in promotion of skilled birth attendant and health facility use since they are the most trusted when it comes to childbirth. Competency of the health care provider is under question by the community. Thus, effort should be exerted to improve competency of service providers with midwifery knowledge and skills and making health facility ready all the time for service delivery (Equipment and materials, service providers). We recommend further research on quality of service provided for laboring mothers.
